# Complete mitochondrial genome sequence of Bekko Tombo *Libellula angelina* Selys, 1883 (Odonata: Libellulidae)

**DOI:** 10.1080/23802359.2019.1624216

**Published:** 2019-07-11

**Authors:** Iksoo Kim, Su Yeon Jeong, Min Jee Kim

**Affiliations:** Department of Applied Biology, College of Agriculture and Life Sciences, Chonnam National University, Gwangju, Republic of Korea

**Keywords:** Mitochondrial genome, Odonata, *Libellula angelina*, Libellulidae, endangered species, phylogeny

## Abstract

The dragonfly *Libellula angelina* Selys, 1883 (Odonata: Libellulidae) has been listed as a critically endangered species by the International Union for Conservation of Nature (IUCN) and is also an endangered insect in South Korea. We sequenced the whole genome (15,233 bp) of *L. angelina* species, which included a set of typical genes and one major non-coding AT-rich region with an arrangement identical to that observed in most insect genomes. The A + T-rich region harbored one identical repeat composed of 65 bp and two tRNA-like structures (*trnF* and *trnK*-like sequences) with proper anticodon and clover-leaf structures. Phylogenetic reconstruction using the concatenated sequences of 13 protein-coding genes (PCGs) and two rRNAs of the representative odonate mitogenomes utilizing both Bayesian inference and maximum-likelihood methods revealed a strong support for the monophyletic Zygoptera and a moderate to high support for the monophyletic Anisoptera suborders. Unlike that in conventional phylogenetic analysis, a relatively strong sister relationship was revealed between the suborders of Anisozygoptera and Zygoptera.

*Libellula angelina* Selys, 1883 (Odonata: Libellulidae), also known as Bekko Tombo is distributed throughout northern China, Japan, and Korea (Inoue 2006; Jung 2012), which is classified as a critically endangered species by the International Union for Conservation of Nature (IUCN), and is also an endangered species in Korea.

An *L. angelina* adult male was collected at Seoun-myeon, Gyeonggi-do, Korea (36°56'17" N, 127°15'44" E) on June 2015 after obtaining the necessary approvals. This voucher specimen was deposited at National Institute of Biological Resources, Incheon, Korea, with the accession number GEIBIN0000339512. DNA was extracted from the hind legs of *L. angelina* species using a Wizard Genomic DNA Purification Kit (Promega, Madison, WI, USA), and four long overlapping fragments (LFs; *COI*-*ND5*, *ND5*-*CytB*, *CytB*-*srRNA*, and *srRNA*-*COI*) were amplified using four sets of primers designed from the available mitogenomes of Odonata (Lee et al. 2009; Lin et al. 2010; Wang et al. 2015); these were then used as templates for primer walking. The *L. angelina* sequence was deposited in GenBank with the accession number MG189907.

We reconstructed the odonate phylogenetic tree using the Bayesian inference (BI) and maximum-likelihood (ML) methods based on the concatenated nucleotide sequences of 13 protein-coding genes (PCGs) and two rRNA genes. The optimal partitioning scheme (6 partitions) and substitution model (GTR + Gamma + I) were determined using the PartitionFinder 2 and the Greedy algorithm (Lanfear et al. 2012, 2014, 2016). BI and ML methods were implemented in CIPRES Portal v. 3.1 (Miller et al. 2010).

The 15,233 bp complete mitogenome of *L. angelina* consisted of two rRNAs, 22 tRNAs, 13 PCGs, and one A + T-rich region. Twelve PCGs had the typical ATN start codon, whereas *ND1* had the atypical TTG codon. Nine of the 13 PCGs had a complete stop codon; however, *COI*, *COII*, *COII*, and *ND5* had incomplete stop codons, i.e. T or TA. The arrangement of this genome was identical to that typically observed in other insects (Cameron 2014).

The A + T-rich region of *L. angelina* was 529 bp. It harboured two identical 55 bp copies, separated by a 57 bp sequence. Additionally, the A + T-rich region of *L. angelina* had two tRNA-like structures: one *trnF*-like structure encoded in the major strand and another *trnK*-like structure, encoded in the minor strand.

Both the BI and ML methods exhibited identical topology. Both the Anisoptera and Zygoptera suborders were monophylies (Figure 1) although the Anisoptera monophyly was poorly supported by ML [bootstrap (BS) = 37%], whereas it was strongly supported by BI [Bayesian posterior probabilities (BPP) = 0.89]. In addition, all the superfamilies (Calopterygoidea and Coenagrionoidea in Zygoptera; and Libelluloidea and Gomphoidea in Anisoptera) and families (Euphaeidae and Calopterygidae in Calopterygoidea; Coenagrionidae in Coenagrionoidea; Libellulidae in Libelluloidea; and Gomphidae in Gomphoidea) were consistently and strongly supported as monophyletic groups. All the analyses consistently supported the sister relationship between the Anisozygoptera and Zygoptera suborders, with moderate to high nodal supports (BPP = 1; BS = 75). The sister relationship between the Zygoptera and Anisozygoptera suborders was unconventional (Rehn 2003; Davis et al. 2011; Kim et al. 2014); however, recent mitogenome-based phylogenetic results consistently supported the sister relationship between these two suborders (Yong et al. 2016; Jeong et al. 2018). Thus, more diverse taxonomic groups might be helpful to correctly infer the odonate phylogeny.

**Figure 1.**
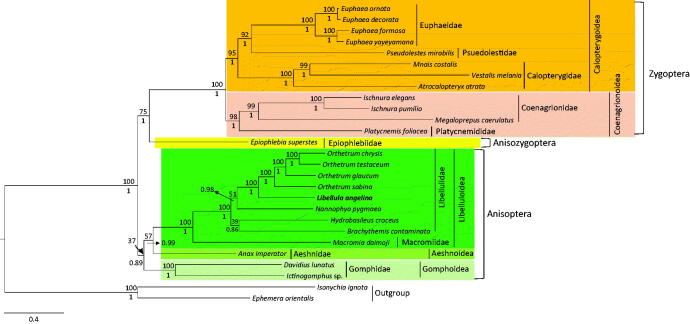
Bayesian inference (BI) method-based phylogenetic tree constructed for the order Odonata using the concatenated sequences of 13 protein-coding genes (PCGs) and two rRNAs. The numbers at each node indicate the bootstrap support using the maximum-likelihood (ML) method (above nodes) and the Bayesian posterior probabilities (BPP; below nodes) using the BI method. The scale bar indicates the number of substitutions per site. Two species belonging to the order Ephemeroptera were used as outgroups. GenBank accession numbers are as follows: *Euphaea ornata*, KF718295 (Cheng et al. 2018); *Euphae decorata*, KF718294 (Cheng et al. 2018); *Euphae formosa*, HM126547 (Lin et al. 2010); *Euphae yayeyamana*, KF718293 (Cheng et al. 2018); *Pseudolestes mirabilis*, FJ606784 (unpublished); *Mnais costalis*, KU871065 (Lorenzo-Carballa et al. 2016); *Vestalis melania*, JX050224 (Chen et al. 2015); *Atrocalopteryx atrata*, KP233805 (unpublished); *Ischnura elegans*, KU958378 (Feindt et al. 2016a); *Ischnura pumilio*, KC878732 (Lorenzo-Carballa et al. 2014); *Megaloprepus caerulatus*, KU958377 (Feindt et al. 2016b); *Platycnemis foliacea*, KP233804 (unpublished); *Epiophlebia superstes*, JX050223 (Wang et al. 2015); *Orthetrum chrysis*, KU361233 (Yong et al. 2016); *Orthetrum testaceum*, KU361235 (Yong et al. 2016); *Orthetrum glaucum*, KU361232 (Yong et al. 2016); *Orthetrum sabina*, KU361234 (Yong et al. 2016); *Nannophya pygmaea*, KY402222 (Jeong et al. 2018); *Hydrobasileus croceus*, KM244659 (Tang et al. 2014); *Brachythemis contaminata*, KM658172 (Yu et al. 2016); *Macromia daimoji* MF990748 (Kim et al. 2018); *Anax imperator*, KX161841 (Herzog et al. 2016); *Davidius lunatus*, EU591677 (Lee et al. 2009); *Ictinogomphus* sp., KM244673 (Tang et al. 2014); *Isonychia ignota*, HM143892 (unpublished); and *Ephemera orientalis*, EU591678 (Lee et al. 2009).
